# Post-transcriptional regulatory patterns revealed by protein-RNA interactions

**DOI:** 10.1038/s41598-019-40939-2

**Published:** 2019-03-13

**Authors:** Andreas Zanzoni, Lionel Spinelli, Diogo M. Ribeiro, Gian Gaetano Tartaglia, Christine Brun

**Affiliations:** 1 Aix-Marseille Univ, INSERM, TAGC, UMR_S1090, Marseille, France; 2 Centre for Genomic Regulation (CRG), The Barcelona Institute of Science and Technology, Dr Aiguader 88, 08003 Barcelona, Spain; 3 Universitat Pompeu Fabra (UPF), 08003 Barcelona, Spain; 4 Institucio Catalana de Recerca i Estudis Avançats (ICREA), 23 Passeig Lluıs Companys, 08010 Barcelona, Spain; 5 CNRS, Marseille, France

## Abstract

The coordination of the synthesis of functionally-related proteins can be achieved at the post-transcriptional level by the action of common regulatory molecules, such as RNA–binding proteins (RBPs). Despite advances in the genome-wide identification of RBPs and their binding transcripts, the protein–RNA interaction space is still largely unexplored, thus hindering a broader understanding of the extent of the post-transcriptional regulation of related coding RNAs. Here, we propose a computational approach that combines protein–mRNA interaction networks and statistical analyses to provide an inferred regulatory landscape for more than 800 human RBPs and identify the cellular processes that can be regulated at the post-transcriptional level. We show that 10% of the tested sets of functionally-related mRNAs can be post-transcriptionally regulated. Moreover, we propose a classification of *(i)* the RBPs and *(ii)* the functionally-related mRNAs, based on their distinct behaviors in the functional landscape, hinting towards mechanistic regulatory hypotheses. In addition, we demonstrate the usefulness of the inferred functional landscape to investigate the cellular role of both well-characterized and novel RBPs in the context of human diseases.

## Introduction

While transcription contributes to coordinated gene expression in time and space, several studies highlighted the discordance between levels of mRNAs and protein production^1,2^. This indicates that the regulation of mRNA transcripts is key to achieve coordinated protein synthesis. Indeed, it has been shown that sets of transcripts coding for functionally related proteins are bound by common regulatory molecules, such as RNA-binding proteins (RBPs) and/or non-coding RNAs, thus forming the so-called RNA regulons^3,4^.

Early protein-RNA interaction mapping studies in yeast demonstrated that many RBPs bind specific mRNAs coding for proteins involved in the same biological process (e.g., ribosome biogenesis, chromatin architecture, oxidative phosphorylation) or that are cytotopically related (e.g., cell wall, endoplasmic reticulum, mitochondrion)^5,6^. In mammalian cells, several sets of related mRNAs are part of RNA regulons as well, *e*.*g*., histone mRNAs bound by the stem-loop binding protein (SLBP)^7^, transcripts involved in inflammation regulated by the RBPs ELAVL1, HNRNPL and TTP^8^, those implicated in DNA damage response and regulated by the RBPs BCLAF1, ELAVL1 and THRAP3^9,10^ and mRNAs coding for cell cycle and proliferation factors bound by Dead end protein homolog 1 (DND1) and Pumilio 1 (PUM1) proteins^9^.

As this regulatory phenomenon has been observed in different species, RNA regulons represent a conserved feature of the post-transcriptional regulation in eukaryotes^3,4,11^. However, even though RNA regulon perturbations can lead to the onset of neurological diseases and cancers in human^12–14^, the control of these regulatory circuits exerted by RBPs is rather sketchy^15,16^, therefore calling for further scrutiny.

A deeper understanding of post-transcriptional regulation is subordinate to the availability of experimentally verified protein-mRNA interaction data. Over the last years, studies based on high-throughput methods to detect RNA molecules bound by RBPs, such as RNA immunoprecipitation and CLIP-based techniques^17,18^ allowed to identify thousands of protein–RNA interactions. However, these studies have focused on the binding ability of a reduced number of established RBPs in a few cell lines^18^, indicating that the protein–RNA interactions space is largely unexplored. Moreover, thanks to the recent development of RNA interactome capture technologies, the catalogue of RBPs has dramatically increased (*e*.*g*.^19–24^). Importantly, many of these RBPs lack a known RNA-binding domain and their role in RNA biology has not been characterized yet^22^. In this context, large-scale computational prediction of protein-RNA interactions can provide a better coverage of the protein-RNA interaction space and improve our understanding of post-transcriptional regulation.

What is the extent of the regulon theory at the coding transcriptome scale? What are the cellular functions regulated at the post-transcriptional level? Can RBPs be classified based on the regulation they exert? To answer these questions, we inferred the functional landscape of the post-transcriptional regulation mediated by the human RBPs, by assessing the RNA regulon theory at different levels of organization of the cellular processes, such as biological pathways and protein complexes. For this, we developed and applied an original large-scale approach to identify cellular processes post-transcriptionally regulated by RBPs, using both experimentally identified and predicted human protein–RNA interactions combined with protein-protein interaction network data and statistical analyses. We showed that the post-transcriptional regulation of functionally-related mRNAs by RBPs concern 10% of the groups that we tested in the regulatory landscape. Furthermore, we identified 3 groups of RBPs possibly regulating these groups of functionally-related mRNAs by using different molecular strategies.

## Results

### A statistical approach to define the human post-transcriptional regulatory landscape

**Figure 1:**
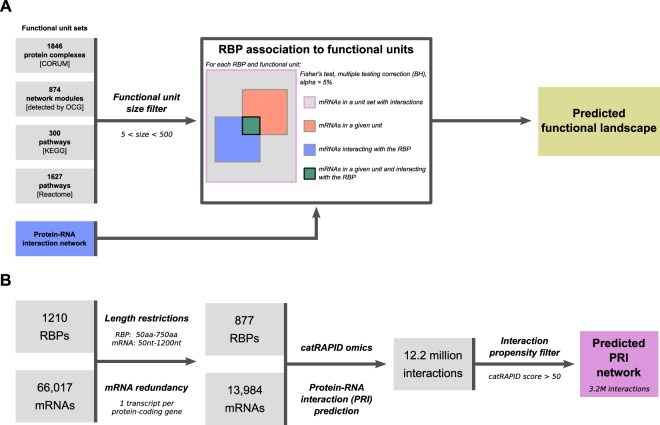
Workflows of our computational strategy. (**A**) General pipeline to test the enrichment and depletion of different functional units in the protein-RNA interaction network to predict the functional landscape of a given RBP. (**B**) Prediction of protein-mRNA interactions (PRI) using the *cat*RAPID *omics* algorithm between experimentally identified human RBPs and a representative set of the human coding transcriptome. The resulting PRI network contains 3.2 million interactions.

We aimed to identify cellular functions that are potentially regulated at the post-transcriptional level by RBPs. According to the regulon theory^3^, an RBP can regulate a given biological process by binding a substantial fraction of mRNAs encoding the proteins involved in that process. We therefore expect to detect a statistically significant over-representation of mRNAs bound by the RBP among groups of functionally-related coding transcripts. To determine the extent of the RNA regulon theory across all human biological processes, we gathered the transcripts encoding proteins involved in the same biological process or pathway, taken from four datasets representing different levels of organization of the cellular functions, and collectively named hereafter “functional units” (FUs): *(i)* 1846 manually curated protein macromolecular complexes from the CORUM database^25^; *(ii)* 874 functional modules detected in a human protein-protein interaction network using the OCG algorithm, which decomposes a network into overlapping modules based on modularity optimization^26^; *(iii)* 300 pathways described in the KEGG database^27^; and *(iv)* 1627 pathways from the Reactome knowledgebase^28^ (Fig. 1A; see Methods). Next, as a proof-of-concept study, we exploited a reduced experimental RBP–mRNA interaction network including 112 RBPs, the interactions of which have been charted using the eCLIP technology^18^ (see Methods). We computed the ratio of interacting *vs*. non-interacting transcripts for each functional unit with every RBP and assessed its significance to be higher or lower than expected by chance by performing a two-sided Fisher’s Exact test (Fig. 1A; see Methods). This strategy allowed us to obtain a broad view on the relationships between RBPs and their functional targets, where a statistically significant over-representation of targets within a functional unit indicates its potential post-transcriptional regulation by the given RBP, and a statistically significant under-representation suggests that certain functional units may avoid the binding of an RBP. By doing so, we interestingly detected three groups of functional units: a first group exclusively enriched in targets of at least one RBP (E-FU units), a second that is both enriched and depleted in RBP targets (M-FU units), and a third group displaying only significant depletions (D-FU units) (Supplementary note, Supplementary Fig. S1A). Concomitantly, we identified two groups of RBPs: one showing only enrichments in targets among functional units (E-RBP set) and a second displaying both significant enrichments and depletions of targets among functional units (M-RBP set). Using our approach, we therefore could classify both RBPs and FUs in distinct groups, based on their behavior in the defined post-transcriptional regulatory landscape. However, the investigated experimental RBP–mRNA interaction network comprises only a portion of the interaction space. Indeed, it is constituted by the protein–RNA interactions identified at large-scale for a subset of well-established RBPs (*i*.*e*., 112) tested in only two cell lines^18^. Since *(i)* we aim at investigating a comprehensive set of RBPs including the non-canonical ones, and *(ii)* the depletion phenomenon observed for both RBPs and FUs could be explained by a lack of coverage of the investigated network, we generalized our analysis to a large computationally predicted network of biophysically possible and biological context-independent interactions between 877 RBPs and 13,984 mRNAs (see below). In doing so, we expectedly circumvented the limitation of our scrutiny by missing interaction data. The results obtained on the experimental RBP–mRNA network (Supplementary Table S1) were used for comparison and assessment purposes.

### A predicted large-scale human RBP-mRNA interaction network

In order to build our RBP-mRNA interaction network, we computed the interaction propensities of 877 experimentally identified human RBPs with a representative set of 13,984 mRNA sequences, covering ~63% of the human protein-coding genes (see Methods), using the *cat*RAPID *omics* algorithm^29^ (Fig. 1B). This tool predicts protein–RNA interactions by exploiting the physicochemical properties of both molecules^30^ and has extensively been used and tested on different RNA and protein datasets with good performances^31–34^, also when compared to other tools (*e*.*g*., in^35^). We generated more than 12 million protein-mRNA interaction predictions, of which 3.2 million show high interaction propensity score (*cat*RAPID score ≥ 50) (see Methods) between the 877 RBPs and ~87% of the initial coding transcripts (12,215 mRNAs). With the standard *cat*RAPID score cutoff^34,36^ (*i*.*e*., interaction propensity ≥ 50), RBPs are predicted to interact with 3176 mRNAs on average (26% of the tested mRNAs) (Supplementary Fig. S2A), *i*.*e*., twice as much as the average number of transcripts found to bind 112 RBPs using the eCLIP technology^18^ when considering the common set of 8028 coding transcripts (Supplementary Fig. S2C). Similarly, *cat*RAPID predicts that mRNAs interact with a higher average number of RBPs (256 RBPs/mRNA, ~30% of the whole set) (Supplementary Fig. S2B) compared to eCLIP detected interactions (8 RBPs/mRNA, 7.5% of the whole set) (Supplementary Fig. S2D). Such differences are expected since *cat*RAPID predictions represent a set of biophysically possible interactions that are independent of the cellular context of the interacting molecules and the experimental conditions in which *in vitro* and *in vivo* studies are carried out. In order to strengthen the confidence in our predictions, we compared the predicted and the experimentally identified interactions using eCLIP for 74 RBPs. Interestingly, for 49 of them, we found an enrichment of experimentally identified binding transcripts among predicted interactors at high interaction propensity score (two-sided Fisher’s Exact test, BH-corrected P-value < 0.05) (Supplementary Table S2).

Overall, to the best of our knowledge, we have generated the largest predicted human RBP–mRNA interaction network to date.

### Statistical enrichments and depletions of RBP binding as an indication of post-transcriptional regulation

Next, we applied our approach (Fig. 1A) to infer the functional landscape of the 877 RBPs. Seven hundred thirteen RBPs (81% of the tested RBPs) showed at least one statistically significant result (5499 in total, BH-corrected P-value < 0.05), namely 3185 significant enrichments (58%) and 2314 significant depletions (42%) involving 300 functional units out of the 2977 tested (Supplementary note, Supplementary Table S3; see Methods). Because some RBPs are predicted to bind many transcripts, we estimated the number of functional units expected to be found over- or under-represented by chance for each RBP with significant results as a control, by randomly shuffling the protein names within the functional units 1000 times (see Methods). All the 713 RBPs passed this test, as their targets were enriched or depleted in a significantly higher number of functional units compared to random. Thus, they were kept for further study (Supplementary Table S3).

**Figure 2:**
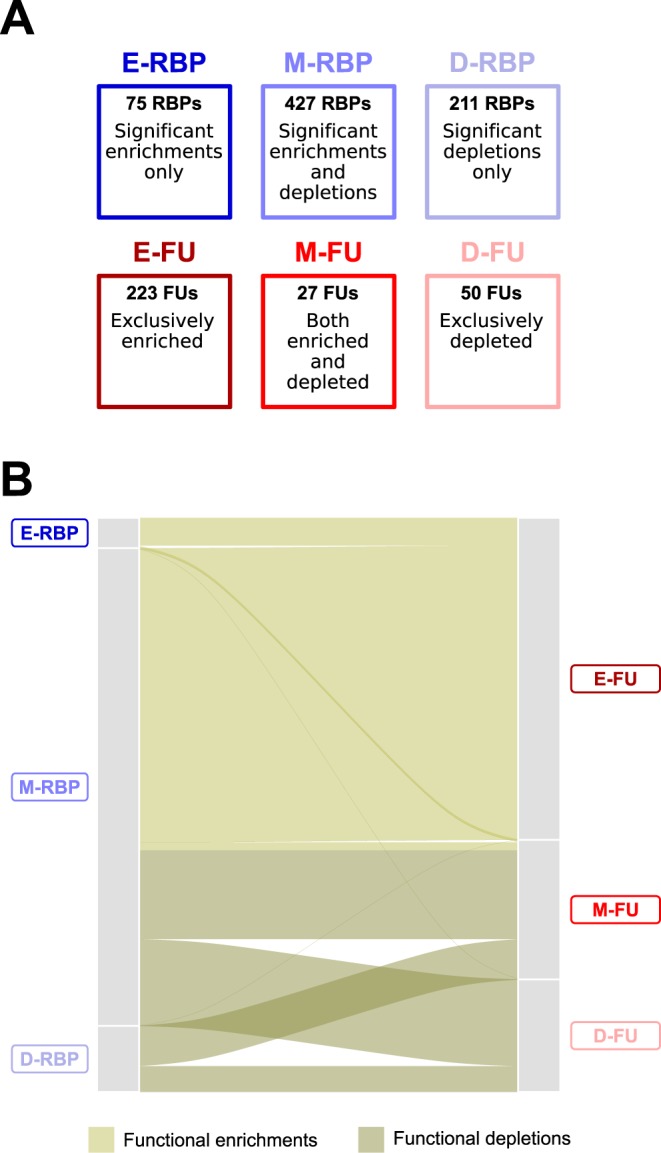
The predicted functional regulatory landscape. (**A**) Summary of the composition of the three RBP (shades of blue color) and functional unit (FU, shades of red color) groups. (**B**) Alluvial plot depicting the functional relationships among RBP and FU groups in the predicted functional regulatory landscape. The thickness of each stream is proportional to the number of enrichments or depletions between two given groups. The size of the grey blocks is proportional to the number of enrichments/depletions in which a given RBP or FU group is involved.

The first important outcome of our functional analysis is that, based on the detection of a significant functional enrichment, we could assign at least one potential target FU to 468 RBPs for which eCLIP interaction data is not available yet (Supplementary Table S3). Second, in accordance with our observations on the experimental RBP–mRNA network (eCLIP-determined mRNA interactors of 112 RBPs, Supplementary note, Supplementary Table S1), our analysis of the predicted RBP–mRNA interaction network reveals an interesting pattern of functional enrichments and depletions. Indeed, it allows grouping RBPs and functional units in three broad categories each (Fig. 2A, Supplementary Tables S4 and S5).

On the one hand, a relatively small number of RBPs only show enrichments in predicted targets among functional units (75 RBPs, ~10% of the RBPs with significant results, named hereafter E-RBP set), indicating that these RBPs exclusively display a binding preference for a number of FUs. A second category accounting for 427 RBPs shows both significant enrichments and depletions of their predicted targets among functional units (~60%, M-RBP set) suggesting that they bind the mRNAs of certain functional units and avoid those of others. Finally, the third category contains 211 RBPs that display only significant depletions (~30%, D-RBP set) within functional units, illustrating that some functional units avoid RBP binding (Fig. 2A,B).

On the other hand, from the perspective of the FUs, we observe a mirrored situation. Most functional units (223 functional units, 74% of the units with significant results, named hereafter E-FU set) are exclusively enriched in targets of at least one RBP, thus possibly regulated at the post-transcriptional level through the binding of those RBPs. Few functional units, namely 27 (9%, M-FU set), are both enriched and depleted in RBP predicted targets, indicating that they may be regulated by the binding of certain RBPs and the avoidance of others. Finally, 50 functional units (~17%, D-FU set) show only significant depletions thereby indicating that their mode of post-transcriptional regulation consists uniquely in the avoidance of RBPs binding (Fig. 2A,B). The comparison with the experimental RBP–mRNA network analysis shows that the three groups of functional units have also been detected (Supplementary Tables S6) with 208 FUs found in both analyses. Importantly, 72% of them display the same relationship with RBPs in terms of E-FU, M-FU and D-FU groups. Finally, the E-RBP and M-RBP sets were also found in the experimental RBP–mRNA network analysis, but none of the analyzed RBP had exclusively depleted functional units among its interactors (*i*.*e*., the D-RBP set). This discrepancy between our predicted regulatory landscape and the results obtained on eCLIP data suggests a possible influence of the chosen *cat*RAPID interaction propensity threshold (*i*.*e*., score ≥ 50).

To assess the extent of this possible impact on the observed enrichment/depletion patterns of the predicted landscape, we carried out a threshold-free statistical analysis based on the GSEA method^37^ (see Methods). Importantly, we found the three distinct categories for both RBPs and functional units, with the M-RBP set being involved in a similar fraction of the significant functional enrichments and depletions (Supplementary Fig. S1B), therefore confirming the observed pattern in the threshold-based predicted functional landscape. However, the fact that the fraction of RBPs in the D-RBP set is lower (9%) (Supplementary note, Supplementary Table S7) when using the threshold-free method compared to the fraction detected by the threshold-based approach (30%), indicates a possible effect of the chosen *cat*RAPID interaction propensity threshold on this specific category, in agreement with its absence when studying the e-CLIP data. Altogether, these assessments show that we chose to favor specificity rather than sensitivity by using strict parameters that limit the occurrence of potential false positives.

Overall, this two-step statistical analysis allowed us to define the potential post-transcriptional regulatory landscape of numerous cellular processes by identifying *(i)* those functional units that can be regulated at the post-transcriptional level and that account for 10% of the tested FUs, and *(ii)* the RBPs responsible for such regulation. The results obtained on both predicted and experimental RBP–mRNA interactions suggest that both FUs and RBPs can adopt several possible regulation strategies and should be classified accordingly.

### The predicted regulatory landscape from the RBP perspective

The classification of RBPs in distinct groups based on the functional analysis of their interactors motivate us to assess whether the RBPs have distinct functional and sequence features as well as system-level properties (Supplementary Table S4).

First, we observed that RBPs in the M-RBP set have a statistically significant higher number of enrichments (average = 6.7, median = 4, P-value = 7.6 × 10^−6^, Mann-Whitney *U* test, one-sided) and depletions (average = 3.9, median = 4, P-value = 7.4 × 10^−13^, Mann-Whitney *U* test, one-sided) compared to those of the E-RBP (average = 3.8, median = 2) and the D-RBP (average = 3, median = 3) sets respectively. This suggests that the more numerous RBP group in our classification (M-RBP set) can potentially regulate the larger number of FUs (Fig. 2B).

Second, we checked whether RBPs of the three groups were characterized by an over-representation of different types of RBPs according to a previously proposed functional classification^22^ (see Methods). Indeed, Beckmann and colleagues annotated RBPs into four classes: (*i*) established RBPs (*i*.*e*., proteins with a known role in RNA biology); (*ii*) RBPs carrying a characterized RNA-binding domain (RBD); (*iii*) enigmRBPs, which are proteins found to bind RNA but lacking a canonical RBD and with no previous evidence of involvement in RNA fate; (*iv*) RNA-binding enzymes, which have an RNA-independent metabolic activity.

**Figure 3:**
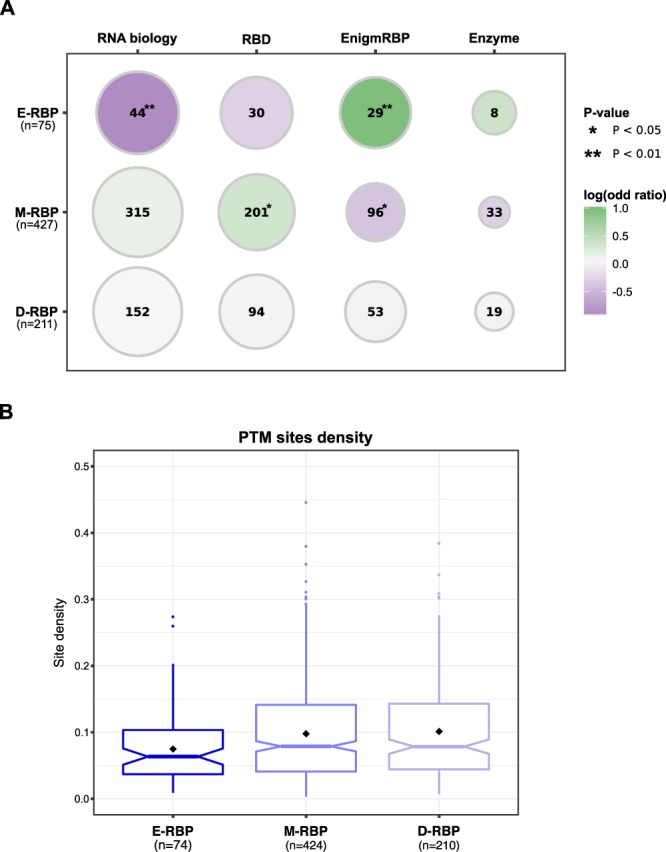
RBPs belonging to the three sets have distinct features. (**A**) Enrichments (circles filled in green) and depletions (circles filled in violet) of different types of RNA-binding proteins among the three groups of RBPs were assessed using the Fisher’s Exact test. Size of the circles is proportional to the fraction of RBPs of a given type that are present in each of the RBP groups, and their frequency is reported as a number within the circle. Significant enrichments and depletions are denoted by one (P-value < 0.05) or two (P-value < 0.01) asterisks. E-RBP: RBPs showing only enrichments in targets among functional units; M-RBP set: RBPs displaying both significant enrichments and depletions of targets among functional units; D-RBP: RBPs display only significant depletions within functional units. (**B**) Distribution of the overall post-translational modification (PTM) density in the sequences of the three RBP groups. Densities for every RBP are computed as the number of experimentally identified PTM sites divided by the RBP sequence length. Black diamonds represent density mean values. Boxplot colors correspond to the RBP group colors in Fig. 2.

We found that established RBPs with a defined role in RNA biology are depleted in the E-RBP set (odds ratio = 0.53, P-value = 0.009, Fisher’s Exact test, one-sided), which is otherwise enriched in enigmRBPs (odds ratio = 2, P-value = 0.004, Fisher’s Exact test, one-sided) (Fig. 3A). In the M-RBP set, we detected a significant over-representation of RBPs with recognized RNA-binding domains (RBDs) (odds ratio = 1.27, P-value = 0.04, Fisher’s Exact test, one-sided) and a significant depletion of enigmRBP (odds ratio = 0.75, P-value = 0.04, Fisher’s Exact test, one-sided). We did not observe any statistically significant over- or under-representation among the D-RBP set. We also checked whether the RBPs in the three groups showed difference in the binding preference of other RNA biotypes based on previous knowledge^38^. Interestingly, we observed that the RBPs binding predominantly mRNAs are more frequent in the E-RBP (82%) compared to the M-RBP (66%) and D-RBP (64%) sets. Indeed, in the latter two sets we observed a higher fraction of ribosomal proteins and RBPs binding small RNAs (Supplementary Table S8). Recent reports showed that many RNA-binding sites of RBPs are found in intrinsically disordered regions^24^ and that RBPs are enriched in low complexity sequence stretches^19^. Hence, we compared the disorder propensity and low complexity content of the RBP sequences belonging to the three different groups using state-of-the-art tools (see Methods). The E-RBP set has a slightly higher disorder (Supplementary Figs S3A and S3B) and low complexity content (Supplementary Fig. S3C) compared to the other two groups. However, these differences are not statistically significant, meaning that these features cannot entirely explain the different enrichment/depletion patterns.

RBPs are generally ubiquitously expressed given their central role in gene regulation^38^. In a compendium of 58 human tissues (see Methods), we did not observe any statistically significant difference among the three groups (Supplementary Fig. S3D), suggesting that the functional enrichment/depletion patterns are independent of the expression breath of the RBPs.

The function of regulatory proteins – such as protein kinases^39^, transcription^40^ and chromatin remodeling factors^41,42^ – is fine-tuned through post-translational modifications (PTMs). Increasing evidence indicates that the activity of RBPs can also be regulated by PTMs^24,43^. We collected the modification site data for seven PTM types from the PhosphoSitePlus database^44^ (see Methods) and mapped them onto the RBP sequences of the three groups. We found that RBPs of the E-RBP set have a significantly lower PTM density (Fig. 3B) compared to M-RBP (Kruskal-Wallis test followed by post-hoc Dunn’s test, corrected P-value = 0.016) and D-RBP (Kruskal-Wallis test followed by post-hoc Dunn’s test, corrected P-value = 0.029) (Supplementary Table S9). When considering individual PTM types alone, a lower density is still observed for the E-RBP set (Supplementary Fig. S4), which is statistically significant for acetylation and phosphorylation (Supplementary Table S9). These results indicate that the function of RBPs belonging to the M-RBP and D-RBP sets can be more finely regulated at the post-translational level than the RBPs of the E-RBP set.

In conclusion, our analyses identified several features discriminating the RBPs belonging to the different groups that could explain the regulatory behavior they may have on functional units.

### The predicted regulatory landscape from the functional unit perspective

A deeper scrutiny of the different behaviors of the FUs shows that the 223 E-FU units are exclusively enriched among the predicted targets of 480 RBPs (average number of RBPs per unit: 13.8), whereas the 50 D-FU units show significant depletions only among the interactors of 499 RBPs (average number of RBPs per unit: 21.5). The 27 functional units in the M-FU groups are enriched among the targets of 74 RBPs (average number of RBPs per unit: 3.7) and depleted among the interactors of 600 RBPs (average number of RBPs per unit: 45.8). These results underline the importance of RBP avoidance as a possible mode of regulation.

What are the cellular processes embodied by the 300 functional units present in the predicted regulatory landscape (Supplementary Table S4)? What are the cellular functions of the potential regulons? E-FU units are involved in processes related to gene expression, such as chromatin organization and regulation, transcription initiation and protein degradation, which are known to be coupled^1,45^. Among the FUs related to chromatin organization and transcription activation, we found SWI/SNF-containing complexes and distinct forms of the Mediator complex from CORUM, as well as several network modules (Supplementary Table S6) and Reactome pathways involved in DNA methylation and RNA Polymerase I transcription initiation. Notably, both SWI/SNF and Mediator complexes have been implicated in RNA processing^46,47^ and their subunit transcripts are regulated post-transcriptionally by miRNAs^48,49^. Moreover, many of these FUs contain histones, whose expression can be controlled at the post-transcriptional level^50^. Altogether, our results underline the role of protein-RNA interactions in coordinating the different steps of gene expression programs, as it has been shown for the regulation of chromatin structure and DNA transcription^51,52^.

Additional enriched FUs are related to cellular processes localized in the mitochondria. Indeed, we find that several FUs have a large number of enrichments, including the large subunit of the mitochondrial ribosome from CORUM, four Reactome pathways related to mitochondrial translation as well as complexes (*e*.*g*., the respiratory chain complex I) and pathways (*e*.*g*., TCA cycle, oxidative phosphorylation) involved in energy production. Interestingly, these results corroborate the known post-transcriptional regulation of the mitochondrial components^53–55^.

M-FU units are involved in several signaling pathways. Indeed, we found that two pathways related to olfactory signaling (one from KEGG and the other from Reactome) are depleted in interactors of around two-third of the tested RBPs. However, they are exclusively enriched in those coded by the ERAL1, G3BP1, G3BP2, MKRN2 and TUFM genes, all expressed in brain tissues, according to Human Protein Atlas^56^ and their coding transcripts have been detected in olfactory sensory neurons (G3BP1, G3BP2, MKRN2, TUFM) or epithelium (ERAL1, TUFM)^57^. Our results indicate that these RBPs could potentially regulate the fate of a regulon made of the olfactory signaling mRNAs.

**Figure 4:**
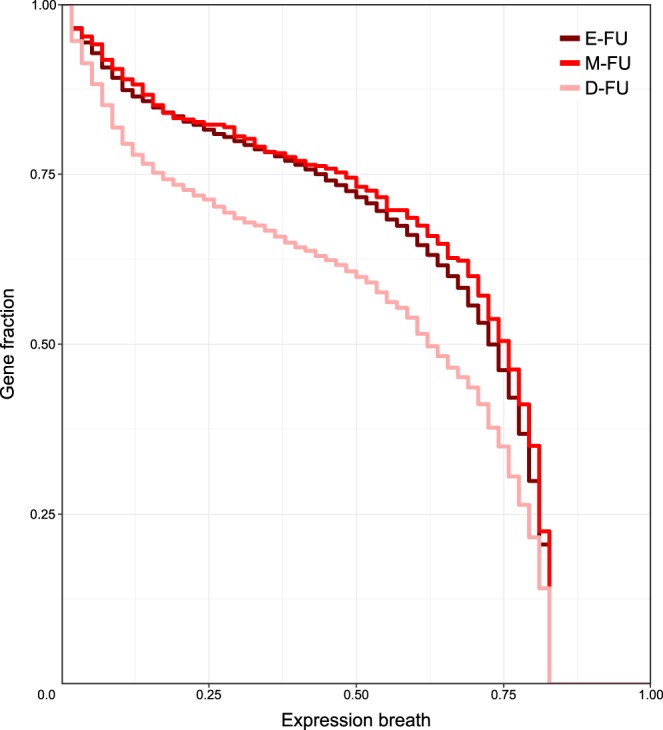
Tissue expression distributions of the proteins annotated in the three FU groups. The color of each distribution corresponds to the FU colors in Fig. 2.

The most frequently depleted units among D-FUs are related to glutamate receptor signaling, defensins and glycosylation of mucins, as well as some units related to cytoskeleton organization. Interestingly, proteins in D-FUs are expressed in a lower number of tissues compared to those in E-FUs (Kolgomorov-Smirnov test, P-value < 2.2 × 10^−16^) and M-FUs (Kolgomorov-Smirnov test, P-value = 1.7 × 10^−10^), respectively (Fig. 4). This suggests that RBP-binding avoidance may participate to the proper tissue-specific expression of the functional unit components.

Finally, 92 (i.e., 71 E-FUs and 5 M-FUs, see Supplementary Table S6) among the 300 FUs were not detected as significantly enriched/depleted in the eCLIP RBP–mRNA network, highlighting the value of protein–mRNA interaction predictions to identify novel potential regulons.

### Disease pathways are targeted by common RBPs

**Figure 5:**
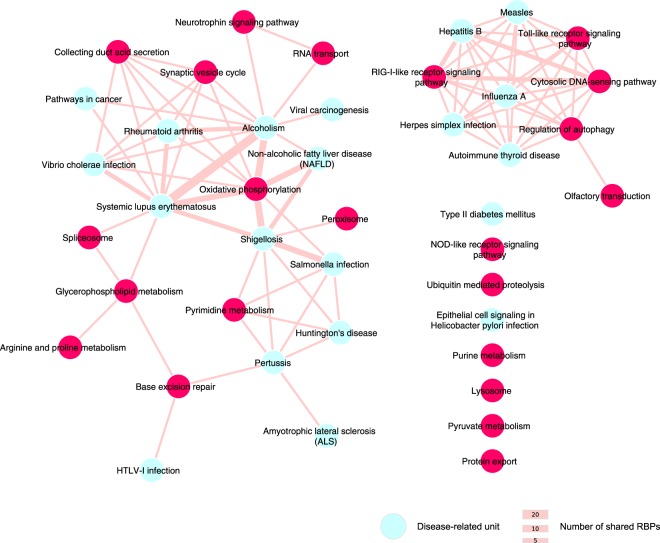
Network representation of disease-related units sharing common RBPs. The size of the edges is proportional to the number of shared RBPs by the two units. Disease units, depicted in cyan, share also RBPs with non-disease related units, depicted in magenta. For sake of clarity, we included only non-disease FUs from the KEGG database.

Among the 223 exclusively enriched functional units (E-FU) we found 20 disease-related pathways from the KEGG database. The majority of them (*i*.*e*., 13) are related to viral and bacterial infections, whereas the other disease functional units are linked to immune-related, neurological and metabolic disorders (Fig. 5). Notably, 17 disease FUs can be regulated by common RBPs, which can also target other non-disease related FUs. For instance, 4 viral infection FUs and one immunological disorder FU are all enriched among the predicted targets of the BTB/POZ domain-containing protein KCTD12, an enigmRBP^22^. KCTD12 predicted interactors are enriched among coding transcripts annotated in three FUs related to immune system pathways (Fig. 5), suggesting that this novel and uncharacterized RBP may be involved in immunity and in infection-related processes.

We also found common RBPs among FUs-related to bacterial infection as well (*i*.*e*., Shigellosis, Pertussis and Salmonella infection pathways). These units are enriched in interactors of the PRKC apoptosis WT1 regulator protein, an RBP encoded by the PAWR gene (also known as PAR4), which it has been implicated in mRNA splicing in cancer cells^58^. Furthermore, the pyrimidine metabolism pathway is also enriched among PAWR predicted interactions. Interestingly, it has been shown that intracellular pathogenic bacteria–such as *Salmonella*, *Shigella* and *Bordetella* (the etiological agent of pertussis)–can modulate several host cell metabolism pathways for their own benefit, including nucleotide biosynthesis^59^, indicating a potential role of PAWR in the post-transcriptional regulation of genes involved in bacterial infection response.

Overall, these results show that our predicted functional landscape is a useful resource to formulate new hypotheses on the cellular role of both established and novel RBPs.

## Discussion

In this work, we explored the post-transcriptional regulation of functionally-related mRNAs by RBPs to, first, estimate the prevalence of the regulon theory at the coding transcriptome scale, and second, detect different behaviors, if present, among the hundreds of RBPs analyzed. As experimentally determined protein–mRNA interactions are still too scarce to allow a large-scale investigation of the post-transcriptional regulation, we computationally predicted an interaction network between representative sets of RBPs and mRNAs to better cover the interaction space. For this, we used *cat*RAPID *omics*, a large-scale protein–RNA interaction predictor that exploits the physicochemical features of the interacting molecules^29,30^, which has been initially validated on a large collection of experimentally identified protein-RNA associations^30–34^. Noticeably, our computational analyses on the *in silico* predicted network have been also performed on available experimentally identified protein–mRNA interactions in order to compare and support all observations.

By studying both types of data, we detected statistically significant over- and under-representations of the mRNAs bound by the RBPs among the functionally related coding transcripts. First of all, these results allow an estimation of the prevalence of the regulon theory. Among the 2977 functional units that we tested, comprising protein complexes, network modules and pathways, 10% have been found possibly regulated in the predicted functional landscapes. This result can be affected by two contrasting factors: *(i)* some FUs may be partially overlapping (*e*.*g*., some protein complexes may play a role in some pathways) or redundant, therefore leading to an overestimation; *(ii)* the choice of a strict *cat*RAPID threshold for the prediction of protein–mRNA interactions, as well as the *cat*RAPID restriction on transcript length, may have led to an under-estimation of the number of potentially regulated FUs. Moreover, as by construction, our statistical approach detects regulation events by considering a pairwise combination of FU and RBP, ignoring possible combinatorial and/or dynamic regulation modulations that could involve several RBPs^16^, the regulon prevalence could have been underestimated. Indeed, the analysis carried out on the eCLIP data provides a higher proportion of regulated FUs (40%, see Supplementary note), thus suggesting that the underestimation is the most plausible scenario.

Second, the different patterns of enrichments and depletions for the RBP binding to functional unit transcripts revealed by our analysis lead to a post-transcriptional landscape shaped by the RBP-mRNA interactions. It reveals that 57,2% (i.e., E-RBP and M-RBP) of the 877 tested RBPs regulate FUs by possibly binding to their mRNAs whereas 72% (i.e., D-RBP and M-RBP) do so by being avoided, therefore indicating the prevalence of this latter RBP regulatory mode. On the other hand, the groups of functionally related mRNAs (the 300 out of 2977 FUs, i.e., 10%) appear to be regulated through binding rather than through avoidance of the RBPs (7.5% enriched in E-FU and M-FU, 2,6% depleted in D-FU and M-FU). Notwithstanding this, 90% of the FUs do not appear as being regulated by a particular RBP. Indeed, promiscuous RBPs interacting with most cellular mRNAs and FUs interacting with those RBPs are not expected to be detected as significantly enriched by our approach since the spread of the RBP targets precludes the detection of a statistically significant signal. This could be the case for 18% of the RBPs (164 RBPs) and 90% of the FUs (2677 FUs) for which no statistical signal has been detected.

We observed 3 different patterns of enrichments and depletions for the RBP binding of functional unit transcripts. These patterns may reflect different possible FU molecular regulation strategies by the RBPs, involving *(i)* the presence of RBP binding in the case of RBP targets enrichment, *(ii)* its avoidance in the case of depletions, or *(iii)* presence or avoidance of binding, when both enrichments and depletions are observed for a given RBP. Indeed, whereas some RBPs (the E-RBP set) appear to act exclusively through their binding to the mRNAs of the FUs (*i*.*e*., presence of binding), some others (the D-RBP set) are excluded from binding by having less targets than expected by chance among the mRNAs of the FUs (*i*.*e*., avoidance of binding). Finally, for other RBPs (the M-RBP set), both strategies, presence and avoidance of binding are observed.

What does the ‘presence’ and the ‘avoidance’ of RBP binding represent? As *cat*RAPID identifies RNA-protein interactions, the ‘presence’ is the physical ability for an RBP to regulate the FUs through its binding, independently of the binding status itself, bound or unbound, which may change with conditions. Conversely, the ‘avoidance’ is the physical inability for the RBP to bind, *e*.*g*., because of the lack of binding sites. As well as the ability, the inability to bind can lead to a regulation event.

Interestingly, the observed depletion, or avoidance of binding, could represent a molecular mechanism that limits inappropriate binding, which could interfere with correct gene expression. Indeed, it has been recently proposed by Savisaar and Hurst^60^ that coding sequences are evolutionarily constrained to avoid certain RBP binding motifs in order to prevent inappropriate interactions that could impair, for instance, their correct mRNA processing. Such avoidance of regulatory elements has also been observed for target sites of microRNAs within 3′UTRs^61^ and to limit spurious transcription binding sites^62^. Our striking observation that some functional units could contain the information to not interact with certain RBPs could therefore represent a cellular regulatory mechanism *per se*, calling for further investigation. However, as the repertoire of experimentally identified mRNA-binding proteins is constantly increasing^63^, we cannot exclude that some of the D-FUs can be regulated by a RBP not present in our dataset.

We further studied the properties of the RBPs belonging to the three sets and found that several features can distinguish them. For instance, the E-RBP set is characterized by an enrichment in enigmRBPs that lack canonical RBDs and for which a role in RNA biology has not been established so far. Among the 29 enigmRBPs in the E-RBP set, there are 8 metabolic enzymes, including the moonlighting protein Leukotriene A-4 hydrolase (LTA4H)^64^ and several signaling and structural proteins. In addition, RBPs in this group have a significant low density in PTM sites, which can regulate, for instance, RNA binding or dictate the subcellular localization of a given RBP^43^. Altogether, this suggests that this set of RBPs contains putative multifunctional proteins whose RNA binding activity, which represents one of their possible molecular tasks, can be potentially modulated by a not yet identified molecular signal.

Conversely, the M-RBP set is enriched in RBPs with canonical RBDs showing a significantly higher PTM density compared to the E-RBP set, consistent with the current knowledge that the function of established RBPs is modulated by post-translational modifications, as in case of SR splicing factors^65^, ELAVL1^66,67^ and FMR1 proteins^68^. Moreover, RBPs in the M-RBP group, as well those in D-RBP, show a wider range of binding preferences among RNA biotypes compared to the E-RBP set, which comprises a high fraction of RBPs binding preferentially/exclusively mRNAs. Overall, our analysis indicates that RBPs in E-RBP group have distinct features that discriminate them from the two other groups. Consequently, further experimental studies are needed to identify the *in vivo* RNA interactors of RBPs in the E-RBP set (only 4 have been tested with the eCLIP technology) and, in the case of the enigmRBPs, decipher their role in mRNA fate.

Altogether, our analyses defined a post-transcriptional regulatory landscape occupied by functionally related mRNA differently regulated by RBPs, thereby allowing us to provide a novel classification of the RBPs. This classification may help understanding the regulatory of action of the continuously increasing number of newly discovered RBPs.

## Methods

### Dataset of experimentally identified protein-RNA interactions

We retrieved interaction information from the ENCODE enhanced CLIP (eCLIP) dataset^18^ gathering 159 experiments for 112 RBPs. We mapped BED peak coordinates referencing the GRCh38 human assembly to Ensembl v82 coding transcript models using BEDTools intersect v2.17^69^ with flag *–wa*. Interactions from replicates and different cell lines were pooled. To have an interaction set comparable to *cat*RAPID predictions, interactions involving transcript isoforms were mapped to the corresponding coding gene and counted as one. Doing so, we obtained a final list of 131,366 experimental interactions between 112 RBPs and at least one transcript encoded by 11,647 genes.

### Compendium of functional units

We built a wide compendium of 4646 functional units and processes by gathering annotations from different sources: 1846 manually annotated human protein complexes from the CORUM database^25^; 873 functional network modules, defined as groups of proteins densely connected through their interactions and involved in the same biological process, detected by the OCG algorithm^26^ on a human protein binary interactome built and annotated as previously described^70–72^ (Supplementary Tables S10–12); 300 maps and 1627 biological pathways from KEGG and Reactome databases, respectively^27,28^. The gene lists annotated in CORUM complexes and biological pathways from KEGG/Reactome were downloaded from the gProfiler webserver^73^ (rev1477, October 2015, based on Ensembl v82), which provides Ensembl identifiers for annotated genes. The genes/proteins annotated in the OCG network modules were mapped to the corresponding Ensembl v82 gene identifiers through the Ensembl BioMart service. We restricted subsequent analyses to complexes, modules and pathways having at least 5 and no more than 500 genes/proteins (*i*.*e*., 2977 functional units).

### Functional unit enrichment analysis on eCLIP interactions

To assess the enrichment/depletion of FU-annotated mRNAs interacting with RBPs in eCLIP dataset, we computed, for each functional unit, the log2-transformed - ratio of FU-annotated mRNAs among RBP interacting and non-interacting transcripts as:

$$ratio=lo{g}_{2}(\frac{\frac{mRN{A}_{fu,int}}{mRN{A}_{fu,int}+mRN{A}_{fu,no-int}}}{\frac{mRN{A}_{no-fu,int}}{mRN{A}_{no-fu,int}+mRN{A}_{no-fu,no-int}}})$$

where mRNA_fu,int_ is the number of FU-annotated mRNAs that interact with a given RBP, mRNA_fu,no-int_ is the number of FU-annotated mRNAs that do not interact with a given RBP, mRNA_no-fu,int_ is the number of mRNAs that interact with the given RBP but that are not present in the FU, and mRNA_no-fu,no-int_ is the rest of mRNAs in the interaction space. We assessed the significance of the enrichment/depletion ratio by performing a two-sided Fisher’s Exact test. P-values were corrected for multiple testing using the Benjamini-Hochberg procedure and we considered as significant only those enrichments/depletions with a corrected P-value below 0.05. We used annotated mRNAs in the eCLIP interaction space as statistical background.

### RNA-binding proteins and coding transcripts

We collected a list of 1217 human RBP protein-coding genes identified by mRNA interactome capture from Beckmann *et al*.^22^ and their corresponding amino acid sequences from the UniprotKB human reference proteome^74^ (May 2016). We downloaded the human coding transcriptome cDNA sequences (66,017 mRNAs) from Ensembl v82^75^ (September 2015).

### RNA-binding protein annotations

For each RBP in our dataset, we gathered from the original article^22^ the following annotations: whether a role in RNA biology is known, presence or absence of a recognized RNA-binding domain according to the classification proposed in Castello *et al*.^19^, whether it has been categorized as ‘classic’ metabolic enzyme (i.e., non-RNA-related enzymes). Those RBPs lacking a recognized RNA-binding and with no established role in RNA biology are labelled as enigmRBP^22^.

### Protein-RNA interaction predictions

We used the standalone version of *cat*RAPID *omics* algorithm^29^, which allows large-scale predictions between transcript and protein sequences, to compute the interaction propensities between human RBPs and coding transcripts. Due to *cat*RAPID computational constraints, we selected mRNA sequences between 50 and 1200 nucleotides of length, as well as protein sequences between 50 and 750 amino acids. Around 72% of the RBPs (877 proteins) and 57% of the human coding transcriptome (37,788 mRNAs) respected the length criterion. To avoid functional biases in subsequent analyses, we further reduced sequence redundancy among mRNAs (i.e., transcript isoforms) by selecting, for each protein-coding gene, the longest transcript as the representative sequence. Doing so, we retained 13,984 transcripts coded by ~63% of the annotated protein-coding genes in Ensembl v82 (22,029 genes). We then predicted more than 12 million protein-RNA interactions between 877 RBPs and 13,984 mRNAs.

### Functional unit enrichment analysis on predicted interactions

To assess the over- and under-representation of the functional units among RBP predicted interactions, as done previously^34,36^, we considered as interacting all RBP-mRNA pairs with a *cat*RAPID interaction propensity score of at least 50 and non-interacting all those with a score below 50. For each functional unit in the compendium, we computed the log2-transformed ratio of the FU-annotated mRNAs among RBP predicted interacting and non-interacting transcripts and assessed its significance as described above for the analysis on eCLIP interaction dataset. As RBPs are predicted to bind to many mRNAs, we further evaluated the number of enrichments/depletions expected by chance in each dataset by shuffling protein labels among functional units 1000 times. Only RBPs having a significantly higher number of enrichments/depletions than expected by chance (empirical P-value < 0.05) were kept.

In a second approach, we carried out a Gene Set Enrichment Analysis^37^ (GSEA) using annotated mRNAs in a given functional unit as gene set. We selected as significant only those enrichments (normalized enrichment score >0) or depletions (normalized enrichment score <0) with a false discovery rate (FDR) < 0.05 based on 1000 gene set permutations. In both tests, we used annotated mRNAs in the *cat*RAPID interaction space as statistical background.

### Intrinsic disorder and sequence complexity

We computed protein residue disorder propensity using the stand-alone version of two state-of-the art disorder prediction algorithms: IUPred^76^ (both long and short predictions) and DISOPRED3^77^. An amino acid was considered disordered if its probability score was greater than 0.4. We calculated the RBP sequence low complexity using the NCBI segmasker application, which is based on the SEG algorithm^78^, using default parameters. For each RBP, we computed the fraction of the number of predicted disordered and low complexity amino-acid residues divided by the sequence length.

### Post-translational modification sites

We collected post-translational modification (PTM) information for 18,030 proteins from PhosphositePlus^44^, which stores data for seven different PTMs: acetylation (20,854 sites in 6874 proteins), methylation (15,195 sites in 5347 proteins), O-GalnAc (2115 sites in 476 proteins), O-GlcnAc (420 sites in 166 proteins), phosphorylation (227,514 sites in 17,464 proteins), sumoylation (7932 sites in 2500 proteins) and ubiquitination (62,256 sites in 10,325 proteins). We extracted PTM data for the RBPs and computed their PTM densities as the number of PTM sites over the sequence length.

### Protein expression profiles

We downloaded protein expression data in human tissues based on immunohistochemistry from the Human Protein Atlas (version 18)^56^. We considered as expressed 10,579 protein-coding genes with a qualitative expression level of at least ‘low’ and a reliability score equal to ‘approved’ or higher. For each protein-coding gene, we computed the expression breath as the fraction of tissues in which the given gene is considered as expressed over the total number of tissues present in the Human Protein Atlas (*i*.*e*., 58).

### Statistical analyses and network visualization

Distributions of disorder propensity and low complexity content fractions, PTM densities and tissue expression breath ratios were compared by using a two-sided Kruskal-Wallis test (significance level = 0.05), a non-parametric analysis of variance method. In case of a null-hypothesis rejection, we applied a *post hoc* Dunn Test, which performs multiple pairwise comparisons between the individual distributions (BH-corrected P-value significance level = 0.05). The network in Fig. 5 was generated using Cytoscape^79^.

## Data Availability

All data generated or analyzed during this study are included in this published article and its supplementary information files. The predicted protein-RNA interactions are available from the corresponding authors on reasonable request.

## Supplementary information

Supplementary Information

Dataset 1
