# Supplementary material for: Post-transcriptional regulatory patterns revealed by protein-RNA interactions
Source: Sci Rep. 2019 Mar 13;9:4302. doi: 10.1038/s41598-019-40939-2 (PMC6416249; doi:10.1038/s41598-019-40939-2)
Supplement: Supplementary file 1 — Supplementary Information [file 41598_2019_40939_MOESM1_ESM.docx]

**Supplementary Information**

**Post-transcriptional regulatory patterns revealed by protein-RNA interactions**

Andreas Zanzoni^1,*^, Lionel Spinelli^1^, Diogo M. Ribeiro^1^, Gian Gaetano Tartaglia^2,3,4^, Christine Brun^1,5,*^

^1^ Aix-Marseille Univ, INSERM, TAGC, UMR_S1090, Marseille, France;

^2^ Centre for Genomic Regulation (CRG), The Barcelona Institute of Science and Technology, Dr Aiguader 88, 08003 Barcelona, Spain;

^3^ Universitat Pompeu Fabra (UPF), 08003 Barcelona, Spain;

^4^ Institucio Catalana de Recerca i Estudis Avançats (ICREA), 23 Passeig Lluıs Companys, 08010 Barcelona, Spain;

^5^ CNRS, Marseille, France.

^*^ Address correspondence to: Andreas Zanzoni ([andreas.zanzoni@univ-amu.fr)](mailto:andreas.zanzoni@univ-amu.fr)) and Christine Brun ([christine-g.brun@inserm.fr)](about:blank).

**Contents**

Supplementary Note

Supplementary Figures S1-S4

# **Supplementary note**

**Functional enrichments and depletions in the eCLIP dataset.** We collected interaction for 112 RBPs from the ENCODE eCLIP dataset (see Methods) and applied the function unit enrichment analysis based on the Fisher’s Exact test as presented in the main text. We obtained 14’660 significant results, namely 13’119 enrichments (89.4% of the total) and 1541 depletions (10.6% of the total) for 99 out of the 112 RBPs. The number of functional units with significant results is considerably higher (i.e., 1339 units, ~45% of the functional units tested) compared to the one obtained based on predicted interactions.

We also observed different patterns of enrichments and depletions for both RBPs and functional units. Indeed, 20 RBPs of them had exclusively enriched functional units among their interactors (E-RBP set) and target almost exclusively functional units in the E-FU set (Figure S2A), whereas 79 showed both functional enrichments and depletions (M-RBP set), with the latter representing only the 11% of the M-RBP significant results. We did not detect any RBP with significant depletion only. We detected the three groups of functional units with distinct enrichment/depletion patterns: 1074 exclusively enriched units (E-FU), 207 depleted only (D-FU) and 58 functional units that were both enriched and depleted (M-FU) and are involved in 407 significant results (Figure S2A), namely 268 enrichments and 133 depletions. The functional annotations of eCLIP interactions are provided in Supplementary Table S1.

**Functional enrichments and depletions in the catRAPID dataset.** As described in the main manuscript, we obtained 5499 significant results, namely 3185 significant enrichments (58%) and 2314 significant depletions (42%) involving 300 functional units out of the 2977 that were tested (Figure 2B in the main manuscript). E-RBP targets chiefly functional units in E-FU set (267 significant enrichments compared to the 24 involving units in the M-FU set), which, in turn, are mainly targeted by M-RBP, namely 2818 functional enrichments representing ~91% of the E-FU relationships with RBPs. Both M-RBP and D-RBP are depleted in functional units belonging to M-FU and D-FU sets. The M-RBP set is one taking part in most of the significant results (85%), as observed in the analysis performed on eCLIP interaction data (Figure S2A).

**An inferred post-transcriptional regulation landscape generated by GSEA.** The choice of interaction propensity threshold (*i.e.*, catRAPID score >=50) used to define the positive and negative predicted interactions sets was based on our previous work (*e.g.*, ^1,2^). However, to exclude that the chosen score threshold could affect our results based on the Fisher's Exact test, we also performed a threshold-free statistical assessment. For each RBP, we ranked predicted interactors according to their propensity score (*i.e.*, from high to low) and we tested the functional units for enrichment, or depletion, using the gene set enrichment analysis (GSEA) algorithm^3^. We obtained 33’603 significant results (27’270 enrichments and 6333 depletions) for 876 RBPs, being the hepatoma-derived growth factor protein, coded by the gene HDGF, the only one with no significant enrichments nor depletions. The number of detected enriched and depleted functional units is twice as much as in the threshold-based test (*i.e.*, 604 functional units compared to 300).

As for the threshold-based predicted functional landscape, we observed a similar enrichment/depletion pattern for RBPs and functional units (Figure S2B). Twenty-four RBPs had exclusively enriched functional units among their predicted targets (E-RBP set). The clear majority of RBPs (*i.e.*, 775, M-RBP set) had both significant enrichments and depletions, representing the majority of the significant results (94%). Only 77 showed only significant depletions (D-RBP set).

We identified three groups of functional units: a predominant subset of exclusively enriched units (*i.e.*, 358, E-FU) and two smaller groups of both enriched/depleted functional units (*i.e.*, 130, M-FU) and exclusively depleted (*i.e.*, 116, D-FU). Finally, around most of the functional enrichments and depletions in the threshold-based predicted functional landscape, 88.5% and 92.4% respectively, were detected as such by the threshold-free analysis.

This comparison, on one hand, confirms the results obtained by the threshold-based approach and, on the other, complements it by expanding the RBP predicted functional landscapes. The GSEA results are provided in Supplementary Table S7.

### **References**

1. Zanzoni, A. *et al.* Principles of self-organization in biological pathways: a hypothesis on the autogenous association of alpha-synuclein. *Nucleic Acids Res.* **41**, 9987–9998 (2013).

2. Ribeiro, D. M. *et al.* Protein complex scaffolding predicted as a prevalent function of long non-coding RNAs. *Nucleic Acids Res.* **46**, 917–928 (2018).

3. Subramanian, A. *et al.* Gene set enrichment analysis: A knowledge-based approach for interpreting genome-wide expression profiles. *PNAS* **102**, 15545–15550 (2005).

**Supplementary Figures**

**Figure S1.** Alluvial plot depicting the functional relationships among RBP (shades of blue color) and FU (FU, shade of red color) groups in (A) the eCLIP interaction dataset and (B) in the GSEA-predicted functional regulatory landscape. The thickness of each stream is proportional to the number of enrichment or depletions between two given groups. The size of the grey blocks is proportional to the number of enrichments/depletions in which a given RBP or FU group is involved.

**
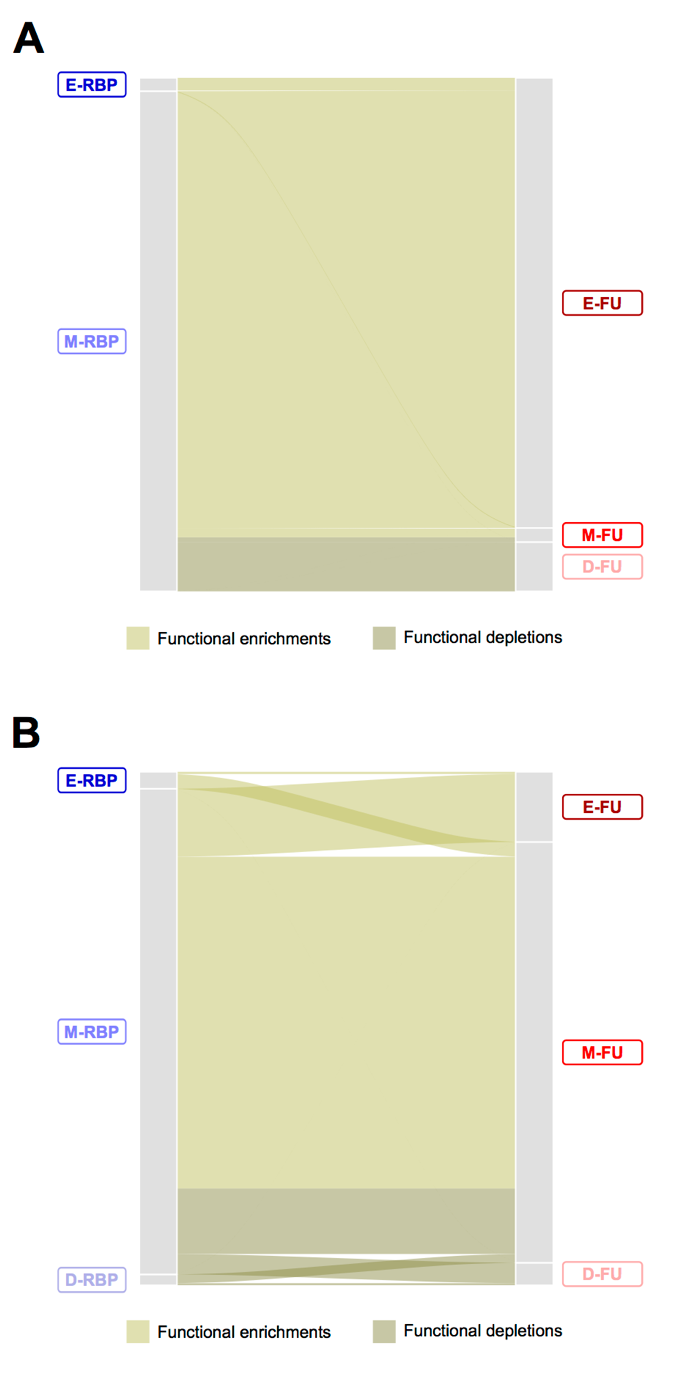
**

**Figure S2.** Distributions of protein-mRNA interactions in the PRI network and eCLIP interaction dataset. (A) Distribution of the numbers of interacting RBP per coding transcript in the PRI network. (B) Distribution of the numbers of interacting mRNA per RBP in the PRI network. (C) Distribution of the numbers of interacting RBPs per coding transcript in the eCLIP interaction dataset. (D) Distribution of the numbers of interacting mRNA per RBP in the eCLIP interaction dataset.

**Figure S3 –** Sequence and functional properties of RBPs. (A) Disorder distributions of the sequences of the three RBP groups based on DISOPRED3 predictions. Disorder content is estimated as the number predicted disordered residues divided by the RBP sequence length. (B) Disorder distributions of the sequences of the three RBP groups based on IUPred ‘long’ predictions. (C) Low complexity distributions of the sequences of the three RBP groups predicted by the SEG algorithm. (D) Tissue expression distributions of the sequences of the three RBP groups based on Human Protein Atlas (HPA) data. Tissue expression is estimated as expression breath, that is the number of tissues in which a given RBP is detected divided by the total number of tissues present in HPA (i.e., 58).

**Figure S4 –** Distribution of the post-translational modification (PTM) density in the sequences of the three RBP groups. Densities for every RBP are computed as the number of experimentally identified PTM sites divided by the RBP sequence length. Black diamonds represent density mean values. Boxplot colors correspond to the RBP group colors in Figure 2. (A) Acetylation. (B) Methylation, (C) Phosphorylation and (D) Ubiquitination. See Table S8 for the statistical results.

# ****
